# Hypoxic vasoconstriction of partial muscular intra-acinar pulmonary arteries in murine precision cut lung slices

**DOI:** 10.1186/1465-9921-7-93

**Published:** 2006-06-29

**Authors:** Renate Paddenberg, Peter König, Petra Faulhammer, Anna Goldenberg, Uwe Pfeil, Wolfgang Kummer

**Affiliations:** 1 University of Giessen Lung Center, Institute for Anatomy and Cell Biology, Justus-Liebig-University, Giessen, Germany

## Abstract

**Background:**

Acute alveolar hypoxia causes pulmonary vasoconstriction (HPV) which serves to match lung perfusion to ventilation. The underlying mechanisms are not fully resolved yet. The major vascular segment contributing to HPV, the intra-acinar artery, is mostly located in that part of the lung that cannot be selectively reached by the presently available techniques, e.g. hemodynamic studies of isolated perfused lungs, recordings from dissected proximal arterial segments or analysis of subpleural vessels. The aim of the present study was to establish a model which allows the investigation of HPV and its underlying mechanisms in small intra-acinar arteries.

**Methods:**

Intra-acinar arteries of the mouse lung were studied in 200 μm thick precision-cut lung slices (PCLS). The organisation of the muscle coat of these vessels was characterized by α-smooth muscle actin immunohistochemistry. Basic features of intra-acinar HPV were characterized, and then the impact of reactive oxygen species (ROS) scavengers, inhibitors of the respiratory chain and Krebs cycle metabolites was analysed.

**Results:**

Intra-acinar arteries are equipped with a discontinuous spiral of α-smooth muscle actin-immunoreactive cells. They exhibit a monophasic HPV (medium gassed with 1% O_2_) that started to fade after 40 min and was lost after 80 min. This HPV, but not vasoconstriction induced by the thromboxane analogue U46619, was effectively blocked by nitro blue tetrazolium and diphenyleniodonium, indicating the involvement of ROS and flavoproteins. Inhibition of mitochondrial complexes II (3-nitropropionic acid, thenoyltrifluoroacetone) and III (antimycin A) specifically interfered with HPV, whereas blockade of complex IV (sodium azide) unspecifically inhibited both HPV and U46619-induced constriction. Succinate blocked HPV whereas fumarate had minor effects on vasoconstriction.

**Conclusion:**

This study establishes the first model for investigation of basic characteristics of HPV directly in intra-acinar murine pulmonary vessels. The data are consistent with a critical involvement of ROS, flavoproteins, and of mitochondrial complexes II and III in intra-acinar HPV. In view of the lack of specificity of any of the classical inhibitors used in such types of experiments, validation awaits the use of appropriate knockout strains and siRNA interference, for which the present model represents a well-suited approach.

## Background

Acute alveolar hypoxia causes pulmonary vasoconstriction [1]. This hypoxic pulmonary vasoconstriction (HPV) directs blood flow towards well ventilated areas of the lung, and, hence, optimizes gas exchange by matching lung perfusion to ventilation. This principally beneficial reflex may turn into a pathogenetic mechanism under conditions of chronic alveolar hypoxia resulting in pulmonary hypertension characterized by remodelling of the pulmonary vasculature and right ventricular hypertrophy. Studies aimed to elucidate the molecular mechanisms underlying acute HPV identified several candidates that may serve as the initial cellular oxygen sensor(s). These include components of the mitochondrial respiratory chain, non-mitochondrial enzymes generating reactive oxygen species (ROS), and plasmalemmal potassium channels [2]. However, partly conflicting data have been obtained and a consensus has not been reached yet.

Still, it is well accepted that, along the pulmonary vascular bed, there is a marked regional diversity in reactivity to hypoxia [3,4]. In the rat, for example, conduit pulmonary artery rings respond to hypoxia after an initial small constriction with a relaxation below baseline, whereas rings from vessels with less than 300 μm in external diameter respond by a monophasic constriction [3]. Thus, at least part of the observed incoherence of data between studies is likely to be due to investigation of different arterial segments and to the use of different experimental approaches. Hemodynamic studies of perfused lungs [5-7] provide valuable information in that they most closely match the clinical situation, but the differential contributions of the various segments of the pulmonary vascular tree can hardly be discriminated. Electrophysiological and force recordings of isolated pulmonary artery segments or of myocytes dissociated from them are primarily aimed to be conducted on small or resistance vessels. Sizes reported for such vessels isolated from rat lung range from <300μm in external diameter [3] to 490 μm in inner diameter [8]. Arteries of that size are fully muscular and usually accompany the conductive airway in its adventitial sheath, although some supernumerary branches that directly pass to the alveolar region immediately adjacent to the bronchoarterial sheath reach this diameter [9].

Micropuncture techniques of subpleural vessels as introduced by Bhattacharya and Staub [10], however, located the most significant drop in perfusion pressure to much more peripheral vascular segments in many species (for review, see [11]) with a particular sensitivity to hypoxia of the arterial part of the microcirculation [12]. Visualization of rat subpleural microvessels by real-time confocal laser scanning luminescence microscopy localized highest sensitivity to hypoxia to immediate pre-capillary (diameter: 20–30 μm) vascular segments [4]. Along the course of smallest pulmonary arteries towards the alveolar capillaries, their muscle coat first becomes incomplete with smooth muscle cells typically being present as a spiral before they vanish and are replaced by a discontinuous layer of intermediate cells and, finally, by pericytes [13,14]. These non-muscular, partially muscular and the thinnest muscular arterial segments are located within the pulmonary acinus, i.e. the region beyond the terminal bronchiolus which represents the basic ventilatory unit [14]. In contrast to the resistance arteries running in the bronchoarterial sheaths, these intra-acinar arteries lack an adventitial layer and are immediately flanked by alveoli whose septa are attached to the vascular elastic lamina. Hence, they are exquisitely located to monitor alveolar oxygen tension within that particular ventilatory unit that they perfuse, and therefore their reactions to reduced oxygen are of particular interest. Anatomically, partial muscular arteries are positioned between the smallest resistance vessels that so far have been dissected for physiological experiments and the surface-near microvessels that can sufficiently be reached by subpleural micropuncture or confocal laser scanning luminescence. Consequently, their responses to changes in oxygen tension have not been directly investigated yet.

In order to bridge that gap we adopted a method that was originally introduced for videomorphometric analysis of airway constriction in precision-cut lung slices (PCLS) [15] and is principally also suited to monitor vascular reactivity, ROS and NO production, and electrophysiological properties of vascular cells [16-19]. In view of further extension of this technique to genetically engineered mouse strains (cf. [19-21]) we performed this study on murine PCLS. Since the general architecture of the pulmonary vasculature in this species is less known than in rat (cf. [13,14,22]) we first conducted a morphological and immunohistochemical survey study on murine PCLS to briefly characterize pre- and intra-acinar vessels. This enabled us to subsequently identify intra-acinar arteries in the videomorphometric set-up. In a recent PCLS study of murine small (inner diameter: 20–150 μm) vessels we obtained evidence for hypoxia-induced ROS generation and for an essential role of complex II of the respiratory chain in this process [18]. Following this line, we used the newly developed approach to assess the impact of inhibition of respiratory chain complexes and of ROS production on HPV in murine intra-acinar arteries.

## Methods

### Reagents

9,11-Dideoxy-11α,9α-epoxy-methanoprostaglandin F_2α _(U46619; final conc.: 10 μM), nitro blue tetrazolium (NBT; final conc.: 500 nM), diphenyleniodonium (DPI; final conc.: 10 μM), 3-nitropropionic acid (3-NPA; final conc.: 5 mM), thenoyltrifluoroacetone (TTFA; final conc.: 50 μM), antimycin A (AA; final conc.: 3 μg/ml), sodium azide (NaN_3_; final conc.: 1 mM), succinate, fumarate, and malic acid (final conc. of each: 15 mM) were purchased from Sigma Aldrich (Deisenhofen, Germany). Sodium nitroprusside (Nipruss; final conc.: 30 μM) was obtained from Schwarz Pharma GmbH Deutschland (Monheim, Germany).

### Preparation of PCLS of murine lungs

PCLS were prepared according to protocols described by Martin et al. [15] and Pfaff et al. [21]. Briefly, 8–12 weeks old FVB mice (Harlan-Winkelmann, Borchen, Germany) were killed by cervical dislocation and the blood was removed from the pulmonary vasculature by in situ perfusion with 37°C HEPES-Ringer buffer (10 mM HEPES, 136.4 mM NaCl, 5.6 mM KCl, 1 mM MgCl_2_, 2.2 mM CaCl_2_, 11 mM glucose, pH 7.4) containing heparin (250 I.U./ml) and penicillin/streptomycin (1%) via the right ventricle. Subsequently, the airways were filled via the cannulated trachea with 1.5% low melting point agarose (Bio-Rad Laboratories GmbH, Munich, Germany) dissolved in HEPES-Ringer buffer. Lungs and heart were removed *en bloc *and transferred into ice-cold HEPES-Ringer buffer to solidify the agarose. The lungs were cut into 200 μm thick slices using a vibratome (VT1000S, Leica, Bensheim, Germany). The agarose was removed by incubation in phenolred-free minimal essential medium (MEM) continuously gassed with 21% O_2_, 5% CO_2_, 74% N_2 _for at least 2 h at 37°C.

### Immunohistochemistry of PCLS

For examination of the morphology of pulmonary vessels, 200 μm thick PCLS were cut from the left lobe of 4 animals as described above except for two changes in the protocol. 1) The lungs were filled with 3% low melting point agarose to facilitate cutting of bronchi that were oriented longitudinally to the cutting direction. 2) The sections were cut in the frontal plane to visualize better the course of blood vessels. After removing the agarose, the sections were fixed for 20 min in ice-cold acetone and washed repeatedly in 0.1 M phosphate buffer. Sections were covered for 1 h with blocking medium (10% normal horse serum, 0.5% Tween 20, 0.5% bovine serum albumin in PBS) followed by overnight incubation with a monoclonal FITC-labelled anti-α-smooth muscle actin (αSMA) antibody (1:500, clone 1A4, Sigma Aldrich). The sections were then washed in PBS and coverslipped in carbonate-buffered glycerol, pH 8.6. The morphology of arteries and veins was evaluated with a Zeiss Axioplan 2 epifluorescence microscope (Jena, Germany).

To assess the morphology of the muscular coat of vessels that were investigated in a videomorphometric analysis, in 23 PCLS the position of the examined vessel was documented at the end of an experiment. Phase contrast images were taken at different magnifications and the slices were subsequently processed for immunohistochemistry as described above. After the labelling, the individual vessel was identified and its α SMA-immunoreactivity was evaluated using either an epifluorescence microscope or a confocal laser scanning microscope (TCS-SP2 AOBS, Leica).

### Videomorphometric analysis of PCLS

Studies on PCLS were performed in a flow-through superfusion chamber (Hugo Sachs Elektronik, March-Hugstetten, Germany) filled with phenolred-free MEM. The slices were held in place in the chamber with nylon strings that were connected to a platinum ring. At the beginning of each experiment the capability of the vessel to contract in response to the thromboxane analogue U46619 and to dilate after application of the NO donor Nipruss was checked. The flow rates were 0.7 ml/min during incubation with normoxic (21% O_2_, 5% CO_2_, 74% N_2_) or hypoxic medium (1% O_2_, 5% CO_2_, 94% N_2_) and 6 ml/min for washing steps. Immediately before feeding the normoxic and hypoxic gassed MEM into the perfusion chamber oxygen partial pressure (pO_2_) was analysed in a blood gas analyser (ABL510, Radiometer, Copenhagen, Denmark). The application of U46619 and Nipruss was performed at flow arrest, the medium containing the other substances was added at low flow rates (0.7 ml/min). The superfusion chamber was mounted on an inverted microscope (Leica), and images of intrapulmonary vessels with inner diameters between 20 and 150 μm were recorded using a CCD-camera (Stemmer Imaging, Puchheim, Germany). Pictures were taken every 2 min using the Optimas 6.5 software (Stemmer Imaging). Changes of the vascular luminal area were evaluated by lining the inner boundaries by hand. The area of the vascular lumen at the beginning of the experiment was set as 100%, and vasoconstriction and -dilatation were expressed as relative decrease or increase of this area.

For a clear graphic presentation of the effects of various substances on hypoxia- and the following U46619-induced pulmonary vasoconstriction, the value obtained immediately before exposure to reduced oxygen was set as 100%. The initial phase of the experiments which tested the viability of the vessels by application of U46619 and Nipruss was not integrated in the graphs unless explicitly stated.

### Statistical analysis

Data are presented as means ± standard error of the mean (SEM) of 5–11 intra-acinar arteries per condition. A single vessel per PCLS was analysed. PCLS for each experimental condition were obtained from 3–5 mice. Statistical analysis was performed using SPSS 11.5.1. Differences among experimental groups were analyzed with the Kruskal-Wallis- followed by the Mann-Whitney-test, with p ≤ 0.05 being considered significant, and p ≤ 0.01 highly significant.

## Results

### α-Smooth muscle actin immunohistochemistry

#### Pulmonary veins

**Figure 1:**
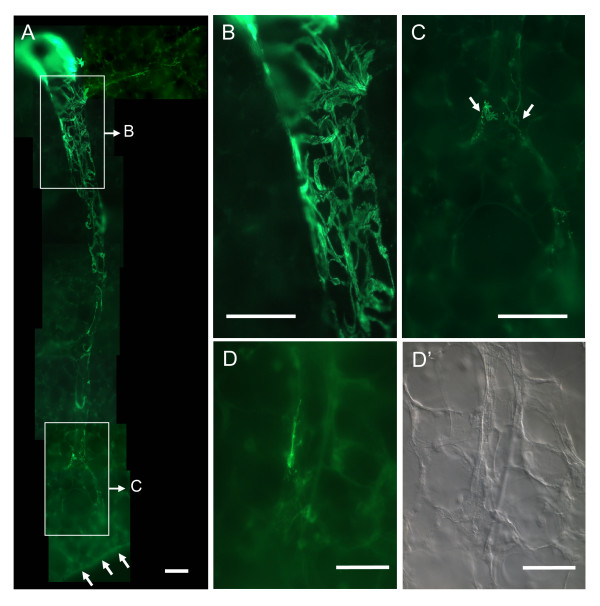
**Distribution of α SMA-immunoreactivity in pulmonary veins**. (**A**) Course of a pulmonary vein from the proximal cardiac muscle covered part (upper left) to the pleural surface (*arrows*). (**B**) Magnification of the vessel shown in upper boxed area in *A*. α SMA-immunoreactive cells are branched and build a loose mesh. (**C**) Magnification of the vessel shown in lower boxed area in *A*. Few immunoreactive cells are present in the distal part of the vein. α SMA-immunoreactive cells are predominantly located close to the branch point (*arrows*). (**D**) A singular, branched α SMA-immunoreactive cell in a distal intra-acinar vein. (**D'**) Differential interference contrast image of *D*. Bars in A, B, C = 100 μm; in D, D' = 50 μm.

Large pulmonary veins were easily identified by their surrounding wall of cardiac muscle cells that were present as continuous autofluorescent sheet surrounding the green fluorescent α SMA-immunoreactive cells. Within this layer of cardiac muscle, strongly α SMA-immunoreactive cells built a loose mesh that continued into branches of the pulmonary vein that were not surrounded by cardiac muscle (Fig. 1A, B). In the distal part of a vein, the meshwork of α SMA-positive cells vanished and the labelled cells lay in small groups or singly within the vessel wall. Immunoreactive cells were located preferentially around branch points (Fig. 1C). In the most peripheral branches of the pulmonary veins, single branched cells were occasionally detectable that exhibited an intensity of α SMA-immunoreactivity comparable to that of cells further proximal in the vessel (Fig. 1D, D').

### Pulmonary arteries and their discrimination from pulmonary veins

**Figure 2:**
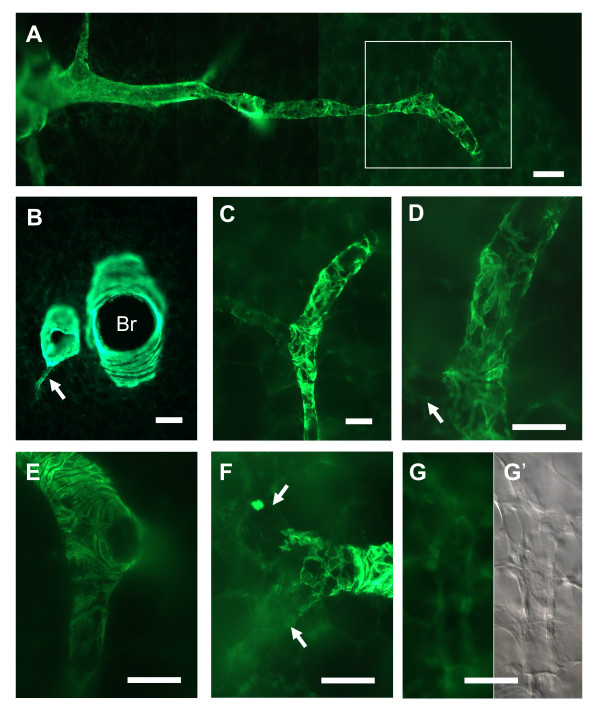
**Distribution of α SMA-immunoreactivity in pulmonary arteries. **(**A**) Course of a pulmonary artery from the bronchus (left) to the pleural surface (right). (**B**) A supernumerary branch (*arrow*) leaves a pulmonary artery that lies close to a bronchus (Br). (**C**) Magnification of the boxed area in *A*. The artery displays a dense mesh of α SMA-immunoreactive cells that becomes wider in distal direction and finally vanishes. To the left, a branch with almost no α SMA-immunoreactive cells leaves the artery. (**D**) Higher magnification from *C *that clearly depicts the branched morphology of α SMA-immunoreactive cells. (**E**) Transition of circular into mesh-like organization of α SMA-immunoreactive cells in the arterial wall. (**F**) At a branch point, the organization pattern of α SMA-immunoreactive cells changes. Only few if any α SMA-immunoreactive cells are visible in the branches (*arrows*). (**G**) Intra-acinar artery devoid of α SMA-immunoreactive cells. (**G'**) Differential interference contrast image of *G*. Bars in A, B = 100 μm; in C-G' = 50 μm.

Large pulmonary arteries ran adjacent to the bronchi. The smooth muscle cells of these arteries showed intense α SMA-immunoreactivity and built a continuous circular layer (Fig. 2A, B). Supernumerary branches that left the main artery without an accompanying division of the bronchi could be identified (Fig. 2B). Regular arterial branches either continued with the same organization of α SMA-immunoreactive cells or displayed a larger mesh with fewer cells as the main branch or proceeded without α SMA-immunoreactive cells (Fig. 2C, E, F). When terminal bronchioli continued into alveolar ducts, the intra-acinar arteries followed the course of the alveolar ducts with a continuous change in the organization of their muscular coat. The continuous circular sheet of α SMA-immunoreactive cells (Fig. 2E) was rearranged into a dense mesh that was frequently organized as a spiral (Fig. 2A). The holes in the mesh became bigger in the distal segments (Fig. 2A, C, D) but, except at a short peripheral segment of the artery (Fig. 2E, F), the mesh was still more densely organized than the wide mesh of pulmonary veins. In the distal arteries, short stretches with a more parallel orientation of cells were observed (Fig 2C, E). This change in orientation was accompanied by a marked reduction of α SMA-immunoreactivity. Further distally, slightly α SMA-immunoreactive cells were only occasionally identified and arterioles continued without α SMA-immunoreactive cells (Fig. 2G, G').

**Figure 3:**
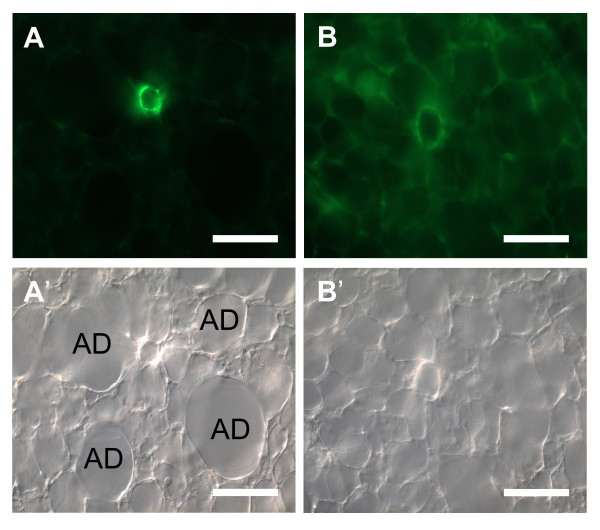
**Difference between arteries and veins in PCLS**. Arteries in cross sections (**A**, **A'**) can be easily distinguished from veins (**B**, **B'**) by their accompanying alveolar ducts (*AD*) in the bright field mode here shown in differential interference contrast (**A'**, **B'**). The muscular coat of arteries is thicker than in veins as revealed by α SMA-immunohistochemistry (**A, B**). Bars = 100 μm.

Large intrapulmonary veins can readily be identified by their sheath of cardiac muscle. Discrimination between arteries and veins is easy if the arteries possess α SMA-immunoreactive cells that are oriented in a dense mesh. Veins, in contrast, do not possess a dense mesh of α SMA-immunoreactive cells but show a wide meshwork or single branched cells in their wall. In addition, in contrast to veins, arteries, with the exemption of supernumerary branches, follow a bronchiolus or run adjacent to alveolar ducts. The only arterial segment that may be difficult to discriminate from veins based on the α SMA-immunoreactivity is the short part that harbors a wide mesh of α SMA-immunoreactive cells or no α SMA-immunoreactive cells at all, if the more proximal parts of the artery are not present in the section. In videomorphometric experiments in which the cells can not be labelled by α SMA-specific antibodies prior to recording of vascular reactivity, cross sections of arteries were distinguished from veins by their thicker tunica media and the close neighbourhood to, usually four, alveolar ducts (Fig. 3).

### PCLS represent a suitable model for studying HPV of partially muscular intra-acinar arteries

**Figure 4:**
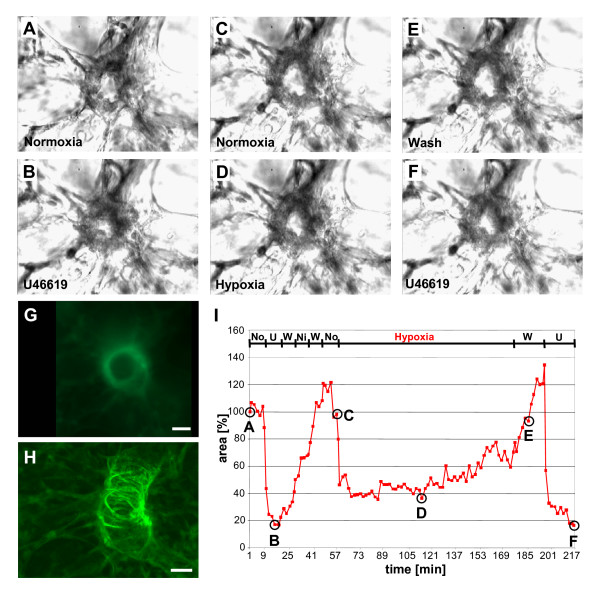
**HPV of a partial muscular intra-acinar artery. **All panels **(A)**-**(I) **refer to the same intra-acinar artery. (**A**) Employing phase contrast optics the cross section of an intra-acinar artery was localized in a PLCS. The vessel responded to U46619 (*U*) with vasoconstriction (**B**), which was reversed by washing the PCLS with normoxic gassed medium (flow rate 6 ml/min) and application of Nipruss. The NO donor was removed by a normoxic wash followed by exposure to normoxic gassed medium (flow rate 0.7 ml/min) (**C**). Perfusion with hypoxic gassed medium induced constriction of the vessel (**D**) which was abolished by washing the PCLS with normoxic gassed medium (**E**). At the end of the experiment, the viability of the artery was validated by application of U46619 (**F**). (**I**) Changes in the luminal areas are expressed as relative values, setting the luminal area at the beginning of the experiment as 100%. Time points for which original micrographs are presented in panels **A-F **are indicated in the curve. *U *= U46619; *W *= wash; *Ni *= Nipruss; *No *= normoxia. After completion of the videomorphometric experiment, the PLCS was stained for αSMA, and the vessel from which luminal diameters were recorded was reidentified (**G**, **H**). Conventional epifluorescence microscopy (**G**) of the 200 μm thick PCLS suggests the presence of a continuous αSMA-immunoreactive muscular coat, but CLSM analysis and three-dimensional reconstruction of the course of this vessel within the PCLS clearly depicts the discontinuous, spiral arrangement of αSMA-immunoreactive smooth muscle cells. Bars = 20 μm.

Small intra-acinar arteries located at gussets of alveolar septa next to alveolar ducts were identified as described above with phase contrast optics in 200 μm thick PCLS. Videomorphometric analysis revealed that the thromboxane analogue U46619 induced an immediate vasoconstriction of these vessels indicated by a reduction of the luminal area to about 40–60% of that observed at the beginning of the experiments. Wash-out of the drug by perfusion with medium and subsequent addition of the NO donor Nipruss induced vasodilatation (Fig. 4). These results demonstrate that intra-acinar arteries maintain their ability to vasoconstriction and -dilatation in PCLS.

To clarify whether these vessels still exhibit HPV, PCLS were exposed to hypoxic gassed medium. The pO_2 _of this medium (gassed previously for about 2 h with 1% O_2_) was reduced to 40 mmHg. We observed an immediately starting, progressive monophasic reduction of the luminal area which reached its maximum after about 20–30 min (40% reduction of the luminal area) and slowly started to fade after 40 min. On average, luminal area returned to initial values after 80 min of exposure to hypoxia, but individual vessels maintained HPV up to 120 min (Fig. 4). Longer periods of hypoxia were not tested. No changes in the luminal area occurred in control incubations with normoxic gassed medium (pO_2_: 160 mmHg). Subsequent incubation of the PCLS with U46619 resulted again in a marked vasoconstriction, regardless whether the PCLS had been previously exposed to normoxic or to hypoxic medium (Fig. 4).

After videomorphometric analysis, PCLS were stained for αSMA as marker for smooth muscle cells. With the aid of low magnification pictures taken at the end of the videomorphometric experiments, vessels were readily re-identified. All of the vessels from which responses to reduced oxygen tension have been recorded before were partially muscular intra-acinar arteries with a relatively thin, discontinuous layer of αSMA-immunoreactive cells typically arranged in a spiral (Fig. 4G, H).

### Intra-acinar HPV requires ROS

**Figure 5:**
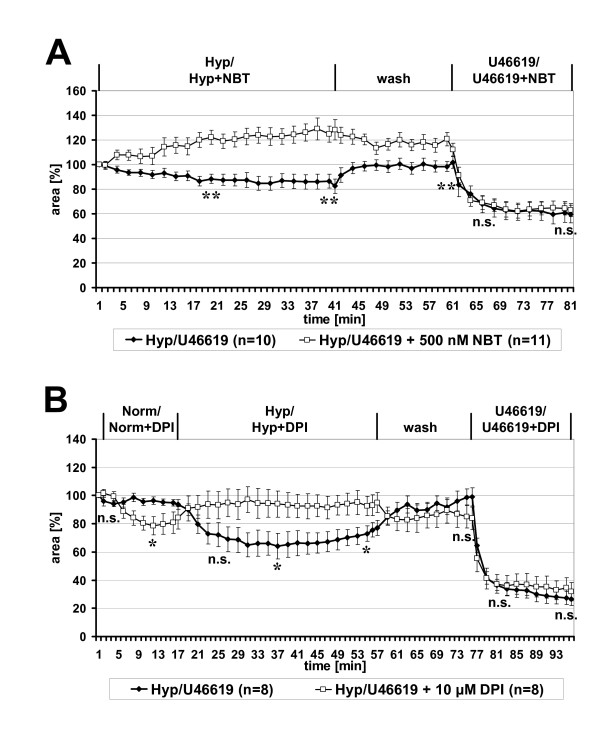
**HPV of intra-acinar arteries requires ROS and flavoproteins. **Videomorphometric analysis of HPV was performed in absence or presence of the ROS scavenger NBT (**A**) and of the flavoprotein inhibitor DPI (**B**), respectively. In both cases, HPV was inhibited whereas U46619-induced vasoconstriction was unaffected. The changes in the luminal areas of the vessels are given as relative values, setting the luminal area immediately before exposure to hypoxia as 100%. Data are presented as means ± SEM. *p = 0.05, ** p = 0.01, n.s. = not significant.

PCLS were incubated with hypoxically gassed medium in absence or presence of the ROS scavenger NBT (Fig. 5A). In this set of experiments exposure to hypoxia induced an about 20% reduction of the luminal area of intra-acinar arteries. In presence of NBT, however, a significant relaxation (20–30% increase in luminal area) of intra-acinar arteries was observed under hypoxia. HPV of the control group was completely and NBT-mediated vasorelaxation was partially reversed by perfusion with normoxic medium, but 20 min after this normoxic perfusion there was still a significant difference between both groups. Application of U46619 diminished in both groups the luminal areas to about 60% of those observed immediately before exposure to hypoxia. These results show that HPV, in contrast to U46619-induced vasoconstriction, requires ROS.

### Intra-acinar HPV requires a flavoprotein

As shown in Fig. 5B, preloading of PCLS with the flavoprotein inhibitor DPI resulted in significant vasoconstriction of intra-acinar arteries under normoxic conditions. This vasoconstriction reversed to dilatation under hypoxia, and HPV was not observed. The capability to respond to U46619 after hypoxic exposure was unimpaired by DPI (Fig. 5B).

### Intra-acinar HPV demands functional mitochondrial complex II

**Figure 6:**
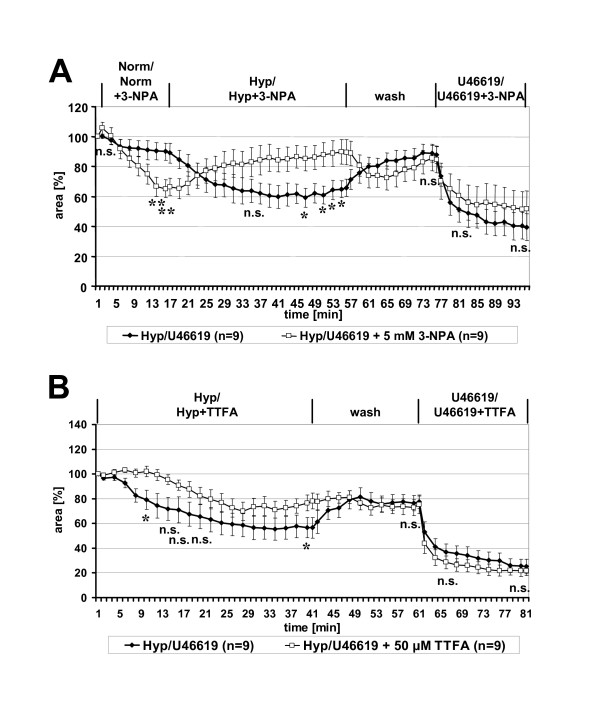
**Involvement of mitochondrial complex II in HPV**. HPV of intra-acinar arteries was analyzed in absence or presence of the complex II inhibitors 3-NPA (**A**) and TTFA (**B**). The response to hypoxia was blocked and decelerated, respectively, in presence of the inhibitors. U46619-induced vasoconstriction was unaffected. Data are presented as means ± SEM. *p = 0.05, ** p = 0.01, n.s. = not significant.

Normoxic preload with 3-NPA, an irreversible inhibitor of complex II subunit SDH-A, induced a rapid and distinct vasoconstriction (Fig. 6A). Switch to perfusion with hypoxic gassed medium cause vasodilatation, and 35 min after onset of hypoxic perfusion the 3-NPA treated vessels significantly differed from those exposed to hypoxia alone (p = 0.001). These differences were abolished by washing the sections with normoxically gassed medium. Subsequent vasoconstrictor responses to U46619 were not significantly different between both groups (Fig. 6A).

Application of another inhibitor of complex II, TTFA, distinctly decelerated HPV (Fig. 6B). Ten minutes after onset of hypoxic exposure, the TTFA-treated vessels still exhibited no vasoconstriction whereas the luminal area of the control group was reduced by 20% (difference between groups: p = 0.024). Thereafter, HPV occurred in the TTFA group with an overall trend towards diminished vasoconstriction compared to the control vessels. The difference between both groups was significant at the end of exposure to hypoxia (p = 0.04). Both groups reacted identically to subsequent application of U46619 (Fig. 6B).

### Succinate, the substrate of succinate dehydrogenase and the product of fumarate reductase activity of complex II, prevents intra-acinar HPV

**Figure 7:**
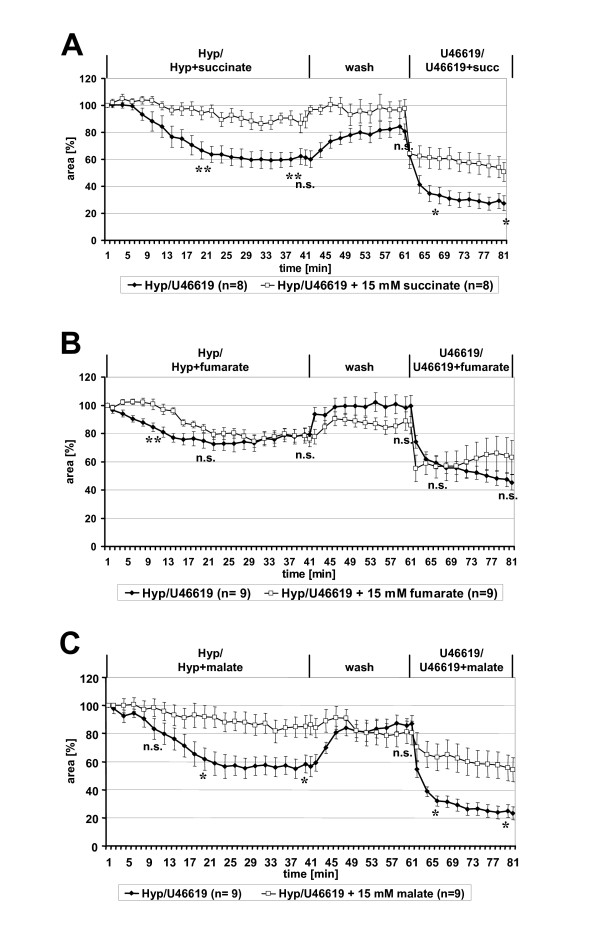
**Effect of the citrate cycle intermediates succinate, fumarate and malate on HPV**. (**A**) Addition of succinate nearly completely blocked HPV. Significant differences between the succinate and the control group in their response to U46619 were noted when the data were standardized to the luminal areas recorded at the beginning of hypoxic exposure, as depicted here, but not when data were standardized to the luminal areas recorded at the end of the post-hypoxic wash period. (**B**) Addition of fumarate delayed the hypoxic response and was without significant effect on U46619-induced vasoconstriction. (**C**) Malate inhibited both HPV and U46619-induced vasoconstriction. Data are presented as means ± SEM. *p = 0.05, ** p = 0.01, n.s. = not significant.

To test whether HPV might be coupled to a functional switch of complex II from succinate dehydrogenase to fumarate reductase we investigated the effects of succinate and fumarate, the substrate and product, respectively of succinate dehydrogenase, and of malate, a potential metabolic precursor of fumarate under hypoxic conditions (Fig. 7). Succinate almost entirely abolished HPV. In presence of succinate, the luminal area was reduced by only 10% under hypoxic conditions whereas a reduction of the luminal area by 40% was observed in the hypoxic control group (Fig. 7A). Subsequent application of U46619 induced distinct vasoconstriction in both groups (reduction of the luminal area of about 60% in the control group and of about 50% in the succinate-treated group). Since, in this particular set of experiments, post-hypoxic vasodilation was incomplete in the untreated group, there were significant differences in the final response to U46619 (time point 66: p = 0.021; time point 80: p = 0.038) when luminal areas were standardized to pre-hypoxic values, but not when responses were standardized to luminal area after the post-hypoxic wash period.

Corresponding experiments with fumarate instead of succinate revealed only weak effects on HPV. The response to hypoxia was delayed, but reached the same degree of vasoconstriction as the untreated control after 30 min (Fig. 7B). Fumarate did not interfere with U46619-induced constriction.

Malate almost entirely prevented HPV, but also diminished markedly the U46619-induced response, indicating general effects of malate on intra-acinar vasoconstriction (Fig. 7C).

### Intra-acinar HPV requires functional mitochondrial complex III

**Figure 8:**
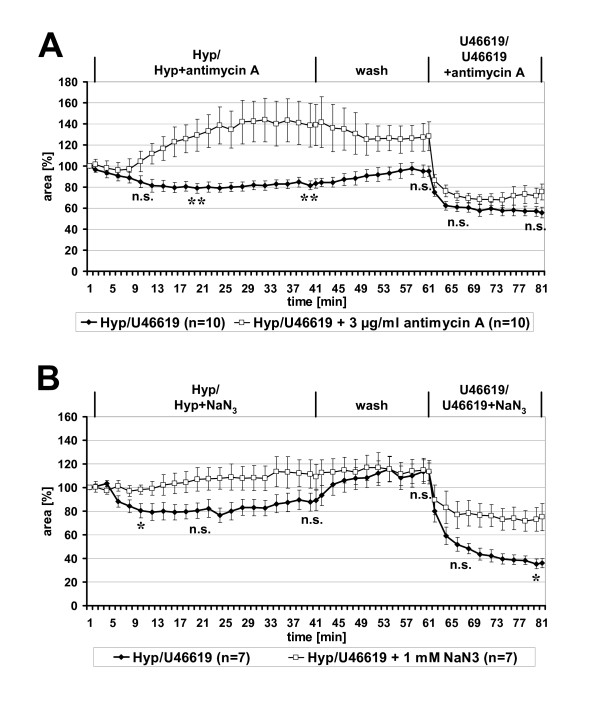
**Specific requirement of mitochondrial complex III, but not of complex IV, for HPV. **Videomorphometric analysis of HPV was performed in absence or presence of the complex III inhibitor antimycin A (**A**) and of the complex IV inhibitor NaN_3 _(**B**), respectively. (**A**) Antimycin A induced distinct vasodilation during hypoxic exposure whereas the response to U46619 was unaffected. (**B**) NaN_3 _inhibited both HPV and U46619-induced vasoconstriction. Data are presented as means ± SEM. *p = 0.05, ** p = 0.01, n.s. = not significant.

Antimycin A, an inhibitor of complex III, caused pronounced vasodilatation instead of constriction in hypoxically gassed medium (Fig. 8A). The luminal area of intra-acinar arteries increased up to 40% after 30 min of hypoxic exposure. Washing of the PCLS with normoxically gassed medium for 20 min partially reversed this dilatation, and subsequent reactivity to U46619 was unimpaired (Fig. 8A).

### Inhibition of complex IV generally affects intra-acinar vasoconstriction

Administration of the complex IV inhibitor NaN_3 _abolished HPV and, instead, intra-acinar vessels exhibited slight dilatation (Fig. 8B). Vasoconstriction induced by U46619 was distinctly diminished by application of NaN_3 _(reduction of luminal area 40% versus 77% in the control group; p = 0.011) (Fig. 8B).

## Discussion

### Intra-acinar murine pulmonary vessels and their constrictor response to hypoxia

With respect to the general structural features that could be evaluated from α SMA-labelled PCLS, murine intrapulmonary arteries show a striking resemblance to that reported for the rat (cf. review by [9]). These similarities include nearly rectangular branching of supernumerary arteries, and step-by-step transition of fully muscular small arteries first to segments where the muscle layer is incomplete with the muscle typically being present as a spiral, and finally to non-muscular segments. Judged from anatomical position, size, and wall thickness, intra-acinar vessels could be identified in living murine PCLS. Despite the fact that they, typically, were only partially muscular they showed a long lasting monophasic constriction in response to hypoxia. In the rat, the partially muscular intra-acinar arteries contain, in addition to the typical vascular smooth muscle cells, so-called "intermediate" cells which lie internal to the elastic membrane. They are considered as contractile cells as well, and as precursors of smooth muscle cells under conditions of vascular remodelling [14,22]. In the immediate precapillary, non-muscular region, intermediate cells are replaced by pericytes which are also interpreted as contractile smooth muscle precursors [23,24]. While data on intermediate cells in the murine lung are lacking, pulmonary pericytes have been identified and reported to be α SMA-negative or stain only weakly at maturity [25,26]. Hence, the distinct HPV of partially muscular intra-acinar arterial segments, i.e. those with incomplete α SMA-immunolabelling, may not only rely on α SMA-positive smooth muscle cells but also on additional contractile cells as well.

Pulmonary artery pressure curves recorded from isolated perfused hypoxic murine lungs reach a peak as early as after 6 min, are biphasic and do not return to basal levels after 3 h even when ventilation is switched to normoxic conditions [27-29]. In contrast, HPV of intra-acinar arteries in PCLS developed slower, was monophasic, and was fully lost after 80 min even at continuing hypoxia. An obvious major difference between these experimental set-ups is the lack of shear-stress acting upon the endothelium in the PCLS model. In perfused vessels, acute HPV leads to a rise in shear-stress thereby inducing secondary mechanisms such as NO release [4,5,30]. Shear-stress alone, however, shall not fully account for the biphasic versus monophasic difference in HPV, because a biphasic response can also be recorded from non-perfused isolated pulmonary artery rings mounted in a vessel myograph (rat: [8]). Still, this type of preparation differs from PCLS in two major aspects beyond that of a species difference: First, it records tension under isometric conditions whereas the latter records metric changes (luminal area) under isotonic conditions, assuming that the force created by stretching the elastic alveolar attachments during vasoconstriction is negligible when the lung is collapsed. Second, resistance vessels analysed by myography, although being small, are still located distinctly upstream to the intra-acinar vessels monitored in PCLS. Thus, it remains to be determined whether a monophasic HPV is an exclusive characteristic of intra-acinar vessels or whether it is also a basic feature of other segments of the pulmonary vasculature when shear-stress and other secondary events are eliminated.

### ROS in intra-acinar HPV

In a preceding study of murine PCLS exposed to hypoxia we observed increased ROS production in small (inner diameter: 20–150 μm) intrapulmonary arteries which required functional complex II of the mitochondrial respiratory chain and was also sensitive to blockade of complex III [18]. The present data provide evidence that these hypoxia-induced events are directly linked to the contractile response of intra-acinar vessels. Evidence for a hypoxia-induced ROS production in pulmonary vessels has been provided earlier by several other approaches (e.g. [31,32]), although there are also conflicting data (e.g. [33]). This controversy is extensively discussed in two recent reviews [34,35], and it is likely that at least part of the inconsistencies between studies is due to investigation of different arterial segments and use of different experimental approaches. With respect to the effects of exogenously applied ROS, e.g. H_2_O_2_, under normoxia and of ROS scavengers under hypoxia, there is less diversity reported: 1) H_2_O_2 _constricts the pulmonary circulation during normoxia [32], 2) inhibition of the endogenous H_2_O_2 _scavenger, catalase, augments HPV [36], and 3) the ROS scavenger NBT prevents HPV in isolated perfused rabbit lungs [37] and completely abrogated intra-acinar HPV in our experiments. Also, DPI, a broad flavoprotein inhibitor that suppresses ROS production in many systems including murine PCLS [18,38], blocks HPV in perfused rabbit lungs [6], pulmonary artery rings from cats [31], and in our murine PCLS model. Hence, the present data are consistent with the view that augmented ROS production is required for HPV of intra-acinar arteries.

### Mitochondrial complexes II and III in intra-acinar HPV

The mitochondrial electron transport chain is currently considered as the major, although not necessarily exclusive, source of hypoxia-induced altered ROS production [34,35]. Particular attention has been paid to complex III which receives electrons from ubiquinol generated either at complex I or complex II. Inhibition of complex III abolishes or attenuates HPV in each model investigated so far [7,8,32,33] including our present study on intra-acinar vessels in murine PCLS. Some uncertainty, however, has arisen as to the specificity of this effect for hypoxia-induced vasoconstriction, since the complex III inhibitor antimycin A also attenuates vasoconstriction elicited by the thromboxane analogue U46619 under conditions of blocked NO synthesis in the isolated perfused rabbit lung [7]. In our model, murine intra-acinar arteries responded to antimycin A with a reversal of HPV to vasodilation although U46619-induced constriction was unimpaired, arguing for a critical role of complex III in HPV of this vascular segment.

The present data provide also evidence for an additional role of complex II in intra-acinar HPV. Complex II is a component of both the Krebs cycle, here also termed succinate dehydrogenase (SDH), and the mitochondrial electron transport chain. It consists of two integral proteins of the inner mitochondrial membrane (SDH-C, SDH-D) that together comprise the heme protein cytochrome b_560_, and two peripheral parts exposed to the mitochondrial matrix, SDH-A and SDH-B [39,40]. The catalytic centre for the enzymatic conversion of succinate to fumarate is located at the flavoprotein SDH-A, and electrons flow along iron-sulphur clusters of SDH-B towards SDH-C/D to, finally, reduce ubiquinon to ubiquinol. There are conditions, however, under which electrons flow in reverse direction so that this complex acts, then, as fumarate reductase [39]. This has been directly shown for isolated mitochondria of the bovine heart and for the rat heart in anoxia or severe hypoxia [41,42]. We have recently obtained evidence for catalytic switch of SDH to fumarate reductase in the course of cellular adaptations to hypoxia in rat sensory neurons and small mouse pulmonary vessels [18,43]. An SDH mutation causes oxidative stress in nematodes [44], and in *Escherichia coli*, where succinate oxidation and fumarate reduction are accomplished by two structurally different enzymes, fumarate reductase is an extremely efficient generator of ROS [45]. Accordingly, complex II activity is essential for hypoxia-induced ROS generation in the pulmonary vasculature [18].

Hence, we have put forward the hypothesis that catalytic switch of complex II to fumarate reductase with concomitant enhanced ROS generation is part of the hypoxia-sensor and -signalling mechanisms in the pulmonary vasculature. Consistent with this concept, succinate, but not fumarate, prevented development of intra-acinar HPV in our present PCLS experiments, which can be explained if complex II acts as fumarate reductase and is inhibited by excess of its reaction product, succinate. It has to be taken into account, however, that succinate and fumarate also exert additional effects. For instance, succinate inhibits prolyl hydroxylases that, in an oxygen-dependent manner, regulate the stability of hypoxia-inducible transcription factor [46], and succinate can also interfere with neurotransmitter receptors [47]. Blockade of HPV by the irreversible inhibitor of the enzymatic centre of SDH-A, 3-NPA, supported the notion of requirement of active complex II for intra-acinar hypoxic constriction. Similarly, inhibition of HPV was achieved by the general flavoprotein inhibitor DPI which also acts upon SDH-A. On the other hand, TTFA, inhibiting complex II by occupying its ubiquinone binding sites [48], was not fully effective in preventing intra-acinar HPV. It has to be taken into account, however, that TTFA also targets extra-mitochondrial enzymes such as esterases [49] so that partly counteracting effects might have been triggered.

Within the respiratory chain, ROS are generated upstream of complex IV at either of the complexes I-III. Thus, the assumption of an involvement of ROS generation in HPV is consistent with the view that HPV is triggered by events located upstream of complex IV. Accordingly, this complex is generally not considered as being specifically involved with HPV, although an unusual cytochrome a3 residing in complex IV has been associated with acute oxygen sensing in the carotid body [50,51] and a recent study also suggests its participation in sustained HPV [52]. In our present experiments, the complex IV inhibitor, NaN_3_, abrogated HPV and also largely reduced U46619-induced constriction. These data argue for an unspecific impairment of intra-acinar vasoconstriction by NaN_3 _rather than for a specific participation of complex IV in HPV. In line with these data, we have recently shown that NaN_3 _also does not block hypoxia-induced ROS generation in smallest intrapulmonary vessels in the mouse [18].

## Conclusion

This study establishes the first model for studying basic characteristics of HPV directly in intra-acinar murine pulmonary vessels, demonstrating a monophasic constrictory response that starts to fade after 40 min. Collectively, the data obtained by various respiratory chain inhibitors and metabolites provide evidence for a critical involvement of the generation of ROS and of mitochondrial complexes II and III in intra-acinar HPV. A general drawback of studies involving classical inhibitors and metabolites, however, is their limited specificity. Since the present model is based upon murine PCLS kept in short-term slice cultures, it provides a promising tool to overcome these problems in future studies by use of genetically engineered mouse strains and siRNA application in vitro.

## Competing interests

The author(s) declare that they have no competing interests.

## Authors' contributions

PK and UP carried out the immunohistochemical characterization of the pulmonary vasculature. RP and WK conceived and designed the videomorphometric analysis of intrapulmonary arteries. These studies were carried out by PF and AG. RP performed the statistical analysis. The manuscript was drafted by RP, PK and WK.
